# Cannabinoid CB1 and CB2 Receptor-Mediated Arrestin Translocation: Species, Subtype, and Agonist-Dependence

**DOI:** 10.3389/fphar.2019.00350

**Published:** 2019-04-10

**Authors:** Mikkel Søes Ibsen, David B. Finlay, Monica Patel, Jonathan A. Javitch, Michelle Glass, Natasha Lillia Grimsey

**Affiliations:** ^1^Department of Pharmacology and Clinical Pharmacology, School of Medical Sciences, Faculty of Medical and Health Sciences, University of Auckland, Auckland, New Zealand; ^2^Centre for Brain Research, Faculty of Medical and Health Sciences, University of Auckland, Auckland, New Zealand; ^3^Department of Pharmacology and Toxicology, University of Otago, Dunedin, New Zealand; ^4^Department of Psychiatry and Pharmacology, Columbia University Vagelos College of Physicians and Surgeons, New York, NY, United States; ^5^Division of Molecular Therapeutics, New York State Psychiatric Institute, New York, NY, United States

**Keywords:** cannabinoid receptor 1 (CB_1_), cannabinoid receptor 2 (CB_2_), G protein-coupled receptor (GPCR), arrestin, cannabinoid, signaling bias, signaling, vasopressin

## Abstract

Arrestin translocation and signaling have come to the fore of the G protein-coupled receptor molecular pharmacology field. Some receptor–arrestin interactions are relatively well understood and considered responsible for specific therapeutic or adverse outcomes. Coupling of arrestins with cannabinoid receptors 1 (CB_1_) and 2 (CB_2_) has been reported, though the majority of studies have not systematically characterized the differential ligand dependence of this activity. In addition, many prior studies have utilized bovine (rather than human) arrestins, and the most widely applied assays require reporter-tagged receptors, which prevent meaningful comparison between receptor types. We have employed a bioluminescence resonance energy transfer (BRET) method that does not require the use of tagged receptors and thereby allows comparisons of arrestin translocation between receptor types, as well as with cells lacking the receptor of interest – an important control. The ability of a selection of CB_1_ and CB_2_ agonists to stimulate cell surface translocation of human and bovine β-arrestin-1 and -2 was assessed. We find that some CB_1_ ligands induce moderate β-arrestin-2 translocation in comparison with vasopressin V_2_ receptor (a robust arrestin recruiter); however, CB_1_ coupling with β-arrestin-1 and CB_2_ with either arrestin elicited low relative efficacies. A range of efficacies between ligands was evident for both receptors and arrestins. Endocannabinoid 2-arachidonoylglycerol stood out as a high efficacy ligand for translocation of β-arrestin-2 via CB_1_. Δ^9^-tetrahydrocannabinol was generally unable to elicit translocation of either arrestin subtype via CB_1_ or CB_2_; however, control experiments revealed translocation in cells not expressing CB_1_/CB_2_, which may assist in explaining some discrepancy with the literature. Overexpression of GRK2 had modest influence on CB_1_/CB_2_-induced arrestin translocation. Results with bovine and human arrestins were largely analogous, but a few instances of inconsistent rank order potencies/efficacies between bovine and human arrestins raise the possibility that subtle differences in receptor conformation stabilized by these ligands manifest in disparate affinities for the two arrestin species, with important potential consequences for interpretation in ligand bias studies. As well as contributing important information regarding CB_1_/CB_2_ ligand-dependent arrestin coupling, our study raises a number of points for consideration in the design and interpretation of arrestin recruitment assays.

## Introduction

Cannabinoid receptor 1 (CB_1_) and cannabinoid receptor 2 (CB_2_) are seven transmembrane domain G protein-coupled receptors (GPCRs). CB_1_ regulates neurotransmission and a range of peripheral functions, whereas CB_2_ regulates immune and inflammatory pathways (Pertwee et al., 2010).

Many cannabinoid ligands demonstrate activity at both CB_1_ and CB_2_. These compounds show a remarkable degree of chemical diversity, and comprise at least four distinct classes: eicosanoids, including all the known endocannabinoids [such as anandamide (AEA) and 2-arachidonoylglycerol (2-AG)]; classical [*Cannabis sativa*-derived Δ^9^-tetrahydrocannabinol (THC) and THC-like]; non-classical (CP55,940); and aminoalkylindole (WIN55,212-2).

The cannabinoid receptors (CBRs) are well-characterized in their signaling via several G proteins to influence the activity of adenylyl cyclase and induce activation of the mitogen-activated protein kinase (MAPK) pathway, among other effects (Felder et al., 1995; Glass and Northup, 1999; Stamer et al., 2001; Bash et al., 2003; Finlay et al., 2017; Ibsen et al., 2017). However, they are less characterized as β-arrestin-coupled receptors. Two β-arrestins are relevant to CB signaling: β-arrestin-1 and β-arrestin-2. It is believed that the downstream effects of β-arrestin-coupled signaling can be isolated from the G protein-mediated pathways and that the non-canonical β-arrestin signaling pathways may be exploitable in the future by the development of novel pharmaceutical agents (Donthamsetti et al., 2018). For this reason, the ability of different agonists to preferentially activate specific intracellular signaling pathways over others – known as “functional selectivity” or “biased signaling” – has recently gained increasing attention (Kenakin and Christopoulos, 2013).

The CBRs have long been considered to hold promise as drug targets. However, to date, only cannabis-derived products have reached the market. The μ-opioid receptor (MOR), which activates Gα_i_-type G proteins and recruits arrestins (like both CB_1_ and CB_2_), provides an example of the therapeutic potential of biased agonism. Studies of MOR suggest that activation of G protein-coupled signaling over β-arrestin-coupled signaling leads to fewer adverse effects (Raehal et al., 2005; DeWire et al., 2013; Manglik et al., 2016), and G protein-biased agonists are now in clinical trial (Viscusi et al., 2016; Singla et al., 2017). In the same manner, it is hypothesized that drugs could target the CBRs but only activate the signaling pathways that elicit desirable effects (Ibsen et al., 2017).

Arrestin–GPCR interactions are thought to follow receptor C-terminal or intracellular loop phosphorylation by a G protein-coupled receptor kinase (GRK) after agonist binding (Moore et al., 2007; Shukla et al., 2013; Zhou et al., 2017). The phosphorylation of GPCRs by GRKs has given rise to the “barcode” hypothesis, wherein GRK isoforms differentially recognize various receptor conformations stabilized by different types of agonists and then phosphorylate the receptors on different sites. These distinctive phosphorylation patterns would then form a “bar code,” which may be recognized by a β-arrestin. Thus, a specific agonist will determine the receptor conformation and subsequent repertoire of canonical signaling responses, the intracellular receptor phosphorylation “bar code,” and thereafter the isoform and conformation of the arrestin that is recruited and direct the downstream effectors (Nobles et al., 2011). For example, depending on the particular agonist used to stimulate the β_2_ adrenergic receptor, either GRK2 or GRK6 phosphorylates the receptor at distinct sites resulting in structurally and functionally distinct β-arrestin-2 conformations (Nobles et al., 2011). Recent observations suggest that CB_1_ signaling is also regulated by different GRK isoforms leading to distinct downstream effects (Delgado-Peraza et al., 2016).

Following GRK-mediated receptor phosphorylation, the binding of arrestin has traditionally been thought to mask the domains interacting with G proteins and thereby prevent further G protein-mediated signaling. As such, arrestin signaling has generally been thought to occur in the absence of bound G protein (Shukla et al., 2014; Kang et al., 2015), although this has been disputed (Thomsen et al., 2016). However, arrestin-mediated signaling likely occurs as a result of different ligand/receptor conformation(s) from those involved in Gα interactions (Kenakin and Christopoulos, 2013).

Both β-arrestin-1 and -2 have been reported to be involved in CB_1_ and CB_2_ signaling. The binding of β-arrestin-2 to CB_1_ has been indicated to cause desensitization and internalization (Jin et al., 1999; Daigle et al., 2008a,b). However, only subtle differences in behavior have been observed between wild-type versus β-arrestin-2 knock-out mice following THC treatment, with enhanced analgesia and hypothermia but no change in catalepsy (Breivogel et al., 2008; Nguyen et al., 2012). The same studies suggested enhanced G protein activation in response to THC in some brain regions of the β-arrestin-2 knock-out mice, but, in contrast, a further study on these mice demonstrated decreased efficacy of THC signaling in brain membranes (Breivogel et al., 2013).

Recruitment of β-arrestin-2 to CB_2_ has been demonstrated using the PathHunter DiscoveRx enzyme complementation assay (McGuinness et al., 2009; Dhopeshwarkar and Mackie, 2016; Soethoudt et al., 2016, 2017) and by measuring the translocation of fluorescently tagged β-arrestin-2 to the cell membrane (Atwood et al., 2012; Nogueras-Ortiz et al., 2017). The downstream effects of β-arrestin-2 interacting with CB_2_ are also suggested to cause internalization and desensitization (Chen et al., 2014). CB_2_ undergoes agonist-mediated C-terminal phosphorylation, followed by a decrease in signaling and surface receptor levels (Bouaboula et al., 1999; Derocq et al., 2000; Chen et al., 2014). Furthermore, agonist-mediated internalization of CB_2_ has been widely observed (Carrier et al., 2004; Shoemaker et al., 2005; Grimsey et al., 2011; Atwood et al., 2012).

Some reports have found little-to-no β-arrestin-1 recruitment to CB_1_ (Gyombolai et al., 2013, 2015; Delgado-Peraza et al., 2016). Others find that β-arrestin-1 is recruited to CB_1_ (Laprairie et al., 2014, 2016), a finding that is corroborated by structural studies of β-arrestin-1 interaction with a synthesized CB_1_ C-terminus (Bakshi et al., 2007; Singh et al., 2011). There is only one recent report suggesting β-arrestin-1 recruitment to CB_2_ (Nogueras-Ortiz et al., 2017), whereas a prior study indicated lack of recruitment or functional involvement in internalization (Chen et al., 2014).

There is also limited evidence indicating the downstream effects of β-arrestin-1 binding to the CBRs. Generally, in binding to GPCRs β-arrestin-1 serves as a scaffold for a pathway leading to the phosphorylation of MAPK extracellular signal-regulated kinase (ERK) (DeWire et al., 2007). There is evidence to support this function of β-arrestin-1 after binding to CB_1_ (Flores-Otero et al., 2014; Delgado-Peraza et al., 2016) and CB_2_ (Nogueras-Ortiz et al., 2017).

The interaction between a GPCR and a β-arrestin is often observed via bioluminescence resonance energy transfer (BRET) or enzyme complementation assays. A potential issue with these types of assays is that protein “modules” are typically fused to both the cytoplasmic tail of the receptor and to the arrestin to enable quantification of the interaction using optical techniques. The signaling capacity of these chimeric proteins is not usually verified, and so it is not obvious as to whether the interactions being reported faithfully reflect those of the native receptor and effector. Imaging-based approaches, on the other hand, involve monitoring of the translocation of a fluorescently tagged arrestin by confocal microscopy. This approach offers the advantage that the receptor of interest need not be tagged, but it is highly labor-intensive, and as a result typically very few cells per experimental treatment are sampled. Consequently, it is highly impractical to perform thorough concentration responses utilizing multiple ligands and/or receptors.

Considering these factors, a novel BRET approach has been developed where the receptor remains untagged, and this approach has been recently utilized to study the dopamine receptor D_2_ (D_2_R) (Clayton et al., 2014; Donthamsetti et al., 2015). In this design, the β-arrestin is fused to the BRET donor modified *Renilla* Luciferase (Rluc8), and the BRET acceptor Citrine is fused to a doubly palmitoylated fragment of GAP43, which anchors it to the plasma membrane. As such, the assay output is not a direct measure of the distance between the receptor and β-arrestin but rather the proximity of β-arrestin to the plasma membrane. Hence, the assay is not a β-arrestin recruitment assay and is instead a *translocation* assay. The membrane translocation of β-arrestins can generally be interpreted as recruitment to the receptor. Using the native receptor circumvents the risk of tags fused to the cytoplasmic tail of the receptor introducing aberrant conformational states, and it ensures that receptor signaling and trafficking are unaffected. Furthermore, the assay allows for comparisons of β-arrestin recruitment capabilities between different receptors. This study has utilized this assay (with receptors minimally modified with an extracellular epitope-tag to facilitate measurement of expression) to investigate the ability of CB_1_ and CB_2_ to induce β-arrestin-1 and -2 plasma membrane translocation in response to stimulation with a range of cannabinoid ligands. Furthermore, we have compared the CB_1_- and CB_2_-mediated activity profiles of human and bovine β-arrestins.

## Materials and Methods

### Plasmids and Cloning

We used the human form of all receptor constructs and transiently expressed transgenes to ensure high expression levels. The pplss-3HA-hCB_1_ pEF4a construct has been described previously (Finlay et al., 2017). The use of the preprolactin signal sequence (pplss) chimera of CB_1_ was necessary to ensure sufficient expression levels. The 3HA-hCB_2_ pEF4a construct has been described previously (Grimsey et al., 2011).

The 3HA-hV_2_R pcDNA3.1 construct and 3HA-hD_2_R pcDNA3.1 construct were both purchased from cDNA Resource Center (#AVR020TN00 and #DRD020TN01, respectively; www.cdna.org, Bloomsburg, PA, United States).

The Rluc8-bβ-arrestin-2-Sp1 pcDNA3.1 (bovine β-arrestin-2) and mem-linker-Citrine-SH3 pcDNA3.1 were described in the original papers detailing the β-arrestin translocation assay (Clayton et al., 2014; Donthamsetti et al., 2015). Rluc8-bβ-arrestin-1-Sp1 was synthesized and cloned into pcDNA3.1 commercially, using the restriction sites HindIII and NotI (GenScript, Piscataway, NJ, United States).

The Rluc8-hβ-arrestin-2-Sp1 pcDNA3.1 (human β-arrestin-2) construct was generated by Restriction-Free Cloning (van den Ent and Lowe, 2006). In brief, the entire human β-arrestin-2 gene except the stop codon was amplified by PCR from the originally sourced pcDNA3.1 plasmid (#ARRB200001; cDNA Resource Center). The primers used for this reaction created overhangs complementary for the proximal Rluc8 and Sp1 regions of the target vector. The reaction product was treated with DpnI (Roche, Basel, Switzerland) to destroy template DNA, and then electrophoresed on a 1% agarose gel and gel extracted. Using the Rluc8-bβ-arrestin-2-Sp1 pcDNA3.1 construct (see above) as a template, a second PCR reaction was performed using the purified PCR product as the primer (with terminal additions complementary for Rluc8 and Sp1 regions) such that the extant bβ-arrestin-2 gene would be replaced with hβ-arrestin-2. This reaction product was DpnI-treated and then transformed into ultracompetent *Escherichia coli*, purified by commercial miniprep kit and sequence verified. Rluc8-hβ-arrestin-1-Sp1 pcDNA3.1 was generated by commercial Gibson Assembly kit (New England Biolabs, Ipswich, MA, United States). In brief, PCR reactions were performed to generate products of: the entire human β-arrestin-1 gene (except the stop codon) with 5′ and 3′ overhangs complementary for the proximal Rluc8 and Sp1 regions of the target vector (amplified from plasmid #ARRB100002, cDNA Resource Center); and the backbone of the target vector, including both Rluc8 and Sp1 fragments but with the original bβ-arrestin-2 fragment removed. Both reaction products were electrophoresed on a 1% agarose gel and gel extracted, and then combined with Gibson Assembly reaction components in a 10 μl final volume. A Gibson Assembly reaction was performed in accordance with the manufacturer’s instructions, and the product was transformed into ultracompetent *E. coli*, purified by commercial miniprep kit, and sequence verified.

The bGRK2 pcDNA3 plasmid was a kind gift from Associate Professor Kevin Pfleger (Harry Perkins Institute of Medical Research, The University of Western Australia, Perth, Western Australia, Australia). Empty pcDNA3.1+ was purchased from Thermo Fisher Scientific (#V79020, Waltham, MA, United States).

### Cell Culture and Transfection

HEK-293 cells (ATCC #CRL-1573, Manassas, VA, United States) were cultured in Dulbecco’s Modified Eagle’s Medium (DMEM) (HyClone, GE Life Sciences, Logan, UT, United States) supplemented with 10% (v/v) fetal bovine serum (FBS) (Moregate Biotech, Bulimba, Australia) in a humidified incubator at 37°C and 5% CO_2_. The transfection protocol was modified from a previously described protocol (Donthamsetti et al., 2015). 4 × 10^6^ HEK-293 cells were seeded in a 10-cm dish and cultured for approximately 24 h in 8 ml of media. For each 10-cm dish to be transfected, 300 ng Rluc8-β-arrestin-Sp1 pcDNA3.1, 12 μg mem-linker-Citrine-SH3 pcDNA3.1, receptor construct (9.6 μg pplss-3HA-hCB_1_ pEF4a, 9.6 μg 3HA-hCB_2_ pEF4a, 2.4 μg 3HA-hV_2_R pcDNA3.1, or 4.8 μg 3HA-hD_2_R pcDNA3.1), and either 0 or 6 μg bGRK2 pcDNA3.1 were combined. Empty pcDNA3.1 vector (without an insert) was added to a total mass of 27.9 μg DNA. The DNA was mixed in Opti-MEM (Thermo Fisher Scientific, Waltham, MA, United States) to a final volume of 500 μl. In a separate tube, 42 μl PEI MAX (1 μg/μl) (Polysciences, Warrington, PA, United States) was mixed with Opti-MEM to a total volume of 500 μl. The 500 μl of DNA in Opti-MEM and 500 μl of PEI MAX in Opti-MEM were mixed (mass ratio of 1:1.5 DNA:PEI MAX) and incubated for 20 min at room temperature (RT). During the incubation the 8 ml of cell media was replaced. The transfection mix was added drop-wise to the 10-cm dish and incubated for approximately 24 h.

### Bioluminescence Resonance Energy Transfer Arrestin Assay

Transfected cells were lifted from the 10-cm dishes by trypsinization and plated in poly-D-lysine (Sigma–Aldrich, St. Louis, MO, United States) coated, white 96-well Costar^®^ plates (Corning, NY, United States) at a density of 6 × 10^4^ cells/well in 100 μl of DMEM with 10% (v/v) FBS and cultured overnight. Cells were equilibrated in Hank’s Balanced Salt Solution (HBSS) (Gibco, Thermo Fisher Scientific, Waltham, MA, United States) supplemented with 1 mg/ml bovine serum albumin (BSA) (ICPBio, Auckland, New Zealand) for 30 min. Coelenterazine H (NanoLight Technologies, Pinetop, AZ, United States) was added to a final concentration of 5 μM for 4 min prior to dispensing drugs and initiating luminescence reading at 475 ± 30 nm (Rluc8) and 535 ± 30 nm (Citrine) on a LUMIstar^®^ Omega luminometer (BMG Labtech, Ortenberg, Germany) to obtain baseline BRET ratios. Stimulating drugs were then added and BRET signals were detected in real-time for 25 min. All drugs (including coelenterazine H) were prepared in HBSS with 1 mg/ml BSA, and all incubations and stimulations were performed at 37°C.

To account for small differences in basal BRET ratios between replicates, post-drug addition data were normalized by subtracting the average individual pre-drug addition (baseline) read. To obtain ΔBRET ratios, the mean vehicle trace was subtracted from each drug condition at matched time points. The 25 min ΔBRET time course data were analyzed using net area under the curve (AUC) analysis (GraphPad Prism v7; GraphPad Software Inc., La Jolla, CA, United States) and the AUC values were used to generate concentration–response curves. All arrestin translocation data therefore have the units of ΔBRET ratio.second (ΔBRET.sec). A subset of the hβ-arrestin-2 experiments were undertaken with a different biosensor expression level than the primary set of experiments. This influenced the overall ΔBRET.sec response magnitude measured. Importantly, response potency was unaffected. Internal controls with equivalent stimulation conditions between the two datasets with different biosensor expression levels were utilized to normalize the data such that they could be meaningfully compared on the same ΔBRET.sec scale. Concentration–response parameters were obtained by fitting three-parameter (Hill coefficient constrained to 1) nonlinear regression curves. No-receptor controls were performed using cells not transfected with receptor (otherwise transfected in the same manner) but stimulated with the same drug concentrations and analyzed in the same way. The no-receptor measurements were subtracted at each concentration point to eliminate non-receptor-mediated effects.

### Drugs

WIN55,212-2 and BAY59-3074 (BAY) were purchased from Tocris Bioscience (Bristol, United Kingdom). CP55,940, AEA, 2-AG, JWH-015, and JWH-133 were purchased from Cayman Chemical Company (Ann Arbour, MI, United States). (-)-*trans*-THC was purchased from THC Pharm GmbH (Frankfurt, Germany). Dopamine hydrochloride was purchased from Sigma–Aldrich (St. Louis, MO, United States). Arginine vasopressin (AVP) was a kind gift from Dr. Mark Oliver (The University of Auckland, Auckland, New Zealand).

Drug stocks were prepared in absolute ethanol (CP55,940, WIN55,212-2, AEA, 2-AG, THC, BAY, JWH-133) or DMSO (JWH-015) and were stored in aliquots at -80°C prior to use. Dopamine and AVP were made up in H_2_O immediately prior to use. Drug aliquots used for experiments involving serial dilutions were always single-use. Vehicle controls for serial dilutions were maintained constant within experiments.

### Immunocytochemistry

Transfected cells were lifted from the dish with trypsin, and plated 6 × 10^4^ per well in a poly-D-lysine-treated clear 96-well plate (Nunc, Roskilde, Denmark) and incubated for approximately 24 h. The wells were aspirated and the cells were fixed in 4% (w/v) paraformaldehyde buffered with phosphate pH 7.4 for 10 min. Following fixation, the cells were washed with phosphate buffered saline (PBS). Cells were then treated with 90% (v/v) methanol for 10 min at -20°C. The methanol solution was aspirated and the remaining methanol evaporated for 10 min at RT before the cells were washed in PBS with 0.2% (v/v) Triton X-100 (PBS-T).

The hemagglutinin (HA) tag was detected by incubation with mouse anti-HA IgG (Biolegend, San Diego, CA, United States) diluted 1:1000 in immunobuffer (PBS-T, 1% v/v goat serum, and 0.4 mg/ml Thiomersal) for 3 h at RT with rocking. The primary antibody was aspirated, and the cells were washed in PBS-T. Secondary antibody, Alexa Fluor 647 goat anti-mouse IgG (Thermo Fisher Scientific, Waltham, MA, United States), was diluted 1:400 in immunobuffer, and incubated overnight at 4°C with rocking. Following staining with the secondary antibody, the cells were washed in PBS-T. The nuclei were stained with Hoechst 33258 (4 mg/ml in H_2_O diluted 1:500 in PBS-T) for 30 min at RT with rocking. Following the nuclei staining the cells were washed in 100 μl PBS-T and stored in PBS-T with 0.4 mg/ml Thiomersal. The image acquisition was performed on an ImageXpress Micro XLS (Molecular Devices, Sunnyvale, CA, United States). Quantitative analysis was performed with MetaXpress^®^ software (Molecular Devices, Sunnyvale, CA, United States) using an in-house journal, which measures staining intensity from receptor-positive cells, as previously described (Finlay et al., 2016).

### Data and Statistical Analysis

All statistical analyses were performed using data from at least three biological (independent) replicates. All plots and curve-fits were obtained using GraphPad Prism v7. Data presented in figures are either a single representative experiment (mean ± SD of technical replicates) or mean (±SEM) from independent experiments (as indicated in the figure legends). Parameters noted in the text and recorded in tables are the means from independent experiments (±SEM).

Statistical tests were executed with SigmaPlot (v.13.0.0.83, Systat Software, Chicago, IL, United States). One-way ANOVA were utilized when comparing more than two groups with one independent variable, and two-way ANOVA for more than two groups with two independent variables. Normality and equality of variance assumptions were verified with Shapiro–Wilk and Brown–Forsythe tests, respectively. If these tests did not pass, data were transformed appropriately to satisfy parametric test assumptions. When overall ANOVA results suggested a significant difference, groups were compared with Holm–Šídák *post hoc* tests (either all pairwise, or versus vehicle, as indicated in the section “Results”). In tabular representations, ^∗^ indicates a *P*-value of <0.05.

## Results

### Bovine β-Arrestin-2 (bβ-Arrestin-2) Translocation

**FIGURE 1:**
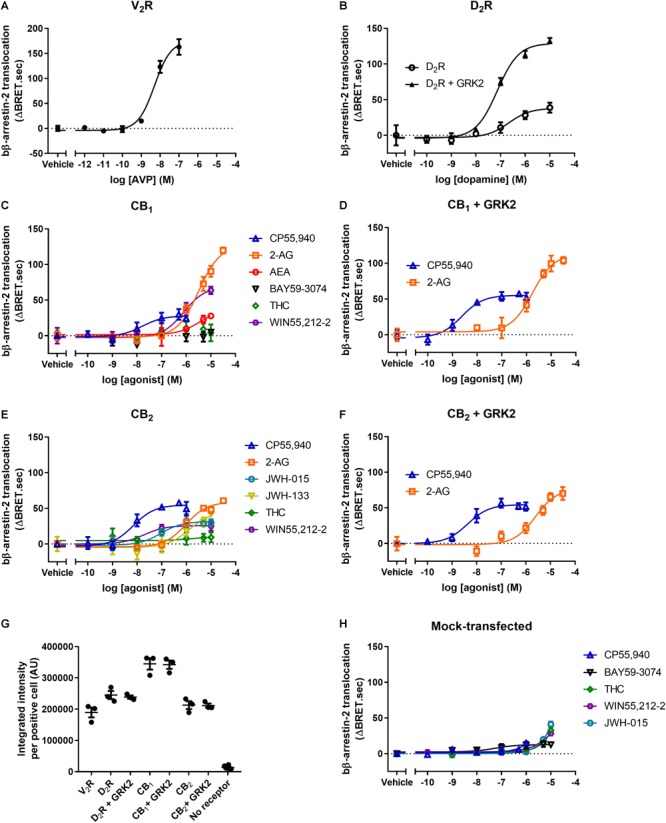
bβ-arrestin-2 translocation to V_2_R, D_2_R, CB_1_, and CB_2_. bβ-Arrestin-2 translocation to plasma membrane in cells expressing **(A)** V_2_R stimulated with AVP, **(B)** D_2_R with or without co-expressed GRK2 stimulated with dopamine, **(C)** CB_1_ stimulated with CP55,940, 2-AG, AEA, BAY, THC, and WIN55,212-2, **(D)** CB_1_ with co-expressed GRK2 stimulated with CP55,940 and 2-AG, **(E)** CB_2_ stimulated with CP55,940, 2-AG, JWH-015, JWH-133, THC, and WIN55,212-2, and **(F)** CB_2_ with co-expressed GRK2 stimulated with CP55,940 and 2-AG. **(G)** Receptor expression of V_2_R, D_2_R, D_2_R+GRK2, CB_1_, CB_1_+GRK2, CB_2_, CB_2_+GRK2, and no-receptor transfected controls in cells used in experiments **(A–G)**. Receptor expression per cell was quantified in cells positive for expression by ICC. **(H)** bβ-Arrestin-2 translocation to plasma membrane in mock-transfected cells. Error bars are ±standard deviation of representative data with three technical replicates **(A–F)**, or standard error of the means from three independent biological replicates **(G,H)**.

We began our investigation using bovine β-arrestin-2 (bβ-arrestin-2). The receptor-independent nature of the β-arrestin translocation BRET assay we employed allows for comparison of β-arrestin translocation between receptors. Human vasopressin receptor 2 (V_2_R) is a known efficient recruiter of β-arrestin-2 (Oakley et al., 1999; Charest and Bouvier, 2003). We used V_2_R expressed in HEK293 cells stimulated with AVP to validate the expression and translocation of bβ-arrestin-2 (Figure 1A). bβ-Arrestin-2 was translocated to the plasma membrane upon V_2_R stimulation with a *p*EC50 of 8.30 ± 0.04, similar to that previously reported (Table 1; Armstrong et al., 2013).

**Table 1:** Bovine β-arrestin-2 translocation to V_2_R and D_2_R.

	bβ-Arrestin-2					
	V_2_R		D_2_R		D_2_ + GRK2	
	logEC50 ± SEM (M)	Emax ± SEM (ΔBRET.sec)	logEC50 ± SEM (M)	Emax ± SEM (ΔBRET.sec)	logEC50 ± SEM (M)	Emax ± SEM (ΔBRET.sec)
AVP	–8.30 ± 0.04	193.6 ± 11.2	X	X	X	X
Dopamine	X	X	–6.85 ± 0.16	40.6 ± 3.1	–7.22 ± 0.13^Δ^	129.0 ± 2.1^Δ^

*X, not determined. ^Δ^Indicates value is significantly different in the presence of GRK2 compared to the absence of GRK2 for the same ligand.*

To investigate bβ-arrestin-2 plasma membrane translocation following CB_1_ activation, we stimulated the receptor with a panel of six well-known CB_1_ agonists: CP55,940, 2-AG, AEA, BAY, THC, and WIN55,212-2 (Figure 1C). CP55,940 was found to be the most potent agonist, while 2-AG was the most efficacious (Table 2). THC and BAY did not elicit a response, indicating that these two ligands do not detectably induce translocation of bβ-arrestin-2 to CB_1_ within the concentration range tested (not significantly different from vehicle at 10 μM, *p* = 0.84; one-way ANOVA).

**Table 2:** Bovine β-arrestin-2 translocation to CB_1_ and CB_2_.

	bβ-Arrestin-2							
	CB_1_		CB_1_ + GRK2		CB_2_		CB_2_ + GRK2	
	logEC50 ± SEM (M)	Emax ± SEM (ΔBRET.sec)	logEC50 ± SEM (M)	Emax ± SEM (ΔBRET.sec)	logEC50 ± SEM (M)	Emax ± SEM (ΔBRET.sec)	logEC50 ± SEM (M)	Emax ± SEM (ΔBRET.sec)
CP55,940	–7.99 ± 0.20	35.2 ± 7.7	–8.45 ± 0.12^Δ^	58.9 ± 9.0	–8.28 ± 0.14	45.4 ± 6.2	–8.44 ± 0.04	70.9 ± 8.7^Δ^
2-AG	–5.33 ± 0.08	116.2 ± 13.3	–5.65 ± 0.09^Δ^	91.7 ± 11.4	–6.10 ± 0.05	45.0 ± 7.1	–5.63 ± 0.09	75.7 ± 8.3^Δ^
WIN55,212-2	–6.15 ± 0.06	72.0 ± 9.2	X	X	–7.71 ± 0.09	25.6 ± 1.9	X	X
AEA	–5.75 ± 0.16	32.1 ± 2.7	X	X	X	X	X	X
THC	NA^∧^	0.54 ± 3.7^∧^	X	X	NA^∧^	2.0 ± 5.2^∧^	X	X
BAY	NA^∧^	2.4 ± 2.8^∧^	X	X	X	X	X	X
JWH-015	X	X	X	X	–6.85 ± 0.11	26.7 ± 2.6	X	X
JWH-133	X	X	X	X	–5.71 ± 0.20	49.7 ± 4.1	X	X

*X, not determined; NA, not active. ^∧^A concentration–response curve could not be fitted, therefore efficacy at 10 μM is noted instead of Emax. ^Δ^Value is significantly different in the presence of GRK2 compared to the absence of GRK2 for the same ligand.*

The β-arrestin translocation BRET assay was initially developed to investigate bβ-arrestin-2 translocation to the human D_2_ dopamine receptor (D_2_R) (Clayton et al., 2014; Donthamsetti et al., 2015). The same work suggests that bβ-arrestin-2 is more efficaciously translocated to D_2_R when GRK2 is over-expressed (Clayton et al., 2014). As the maximum efficacy of translocation of the most efficacious ligand (2-AG) for CB_1_ was only approximately 57% of the vasopressin response, we investigated whether GRK2 potentiates the translocation of bβ-arrestin-2 to CB_1_. Thus, we co-expressed GRK2 and stimulated with CP55,940 and 2-AG (Figure 1D). We chose to utilize only these two ligands because CP55,940 had high potency, while 2-AG had high efficacy. The co-expression of GRK2 did not significantly alter the efficacy of translocation of bβ-arrestin-2 to CB_1_ when stimulating with CP55,940 or 2-AG (CP55,940: *p* = 0.15, 2-AG: *p* = 0.25; two-way ANOVA), however, both ligands appeared to translocate arrestin with slightly increased potency, reaching statistical significance for CP55,950 which was shifted by half a log unit (*p* = 0.036; two-way ANOVA) (Table 2). We verified that we could replicate the prior finding by assaying D_2_R expressed with or without co-expressed GRK2 and stimulated with dopamine. As previously reported, in the presence of co-expressed GRK2, bβ-arrestin-2 was translocated with a significantly greater efficacy (*p* < 0.001; two-way ANOVA) and we also observed that potency was increased (*p* = 0.038; two-way ANOVA) (Figure 1B and Table 1).

To investigate the translocation of bβ-arrestin-2 to CB_2_, we stimulated the receptor with a panel of six well-known CB_2_ agonists: CP55,940, 2-AG, JWH-015, JWH-133, THC, and WIN55,212-2 (Figure 1E). CP55,940 was the most potent agonist at inducing bβ-arrestin-2 translocation via CB_2_. CP55,940, 2-AG, and JWH-133 were similarly and maximally efficacious, while THC was again inactive (not significantly different from vehicle at 10 μM, *p* = 0.72; *t*-test) (Table 2). All ligands with measurable responses were very low efficacy in comparison with AVP at the vasopressin receptor, ranging from approximately 13–26% of the maximum signal observed with this V_2_R.

The effect of co-expressing GRK2 on bβ-arrestin-2 translocation to CB_2_ was assayed following stimulation with CP55,940 and 2-AG (Figure 1F). The co-expression of GRK2 significantly increased the translocation of bβ-arrestin-2 to CB_2_ when stimulating with either ligand (CP55,940: *p* = 0.046, 2-AG: *p* = 0.022; two-way ANOVA) (Table 2).

To confirm receptor expression, and particularly to verify similar expression levels between conditions with and without GRK2, we carried out immunocytochemistry (ICC) and quantified the expression levels (Figure 1G). Reassuringly, the receptor expression levels between conditions with and without GRK2 co-expression were similar. Overall, the expression of the V_2_R, D_2_R, and CB_2_ were very similar, with CB_1_ levels being generally higher. Thus, the low efficacy of interactions between CB_1_ or CB_2_ and the arrestin is unlikely to be due to low receptor expression levels.

For each ligand assayed, bβ-arrestin-2 translocation was also measured in mock-transfected cells lacking the receptor of interest (Figure 1H). Any such “off-target” responses were subtracted from the data discussed above prior to analysis. CB_1_ or CB_2_ mRNA is undetectable in HEK-293 cells (Atwood et al., 2011), and absence of CBRs is also supported by a lack of response with 2-AG (which when CB_1_ and CB_2_ were present induced arrestin translocation with the greatest efficacy of the set of ligands tested; Table 2 and Figure 1C,E). JWH-015, WIN55,212-2, and THC induced the greatest non-CB_1_/CB_2_-mediated responses; JWH-015 and WIN55,212-2 induced translocation similar to the CB_2_-mediated response (i.e., the maximal response in CB_2_-containing cells would have been approximately twice the magnitude shown in Figure 1E prior to subtracting the off-target response), and THC producing a response equivalent to that in CB_1_- or CB_2_-expressing cells (hence our conclusion above that THC does not induce bβ-arrestin-2 translocation to CB_1_- or CB_2_). These non-CB_1_- or CB_2_-mediated responses were all low potency and only significantly different from vehicle at or above 3 μM (*p* = < 0.001–0.003; two-way ANOVA). CP55,940 and BAY also induced small but statistically significant responses at or above 1 μM. The remainder of the ligands studied did not induce any measurable bβ-arrestin-2 translocation in the absence of an introduced receptor (data not shown).

### Human β-Arrestin-2 (hβ-Arrestin-2) Translocation

**FIGURE 2:**
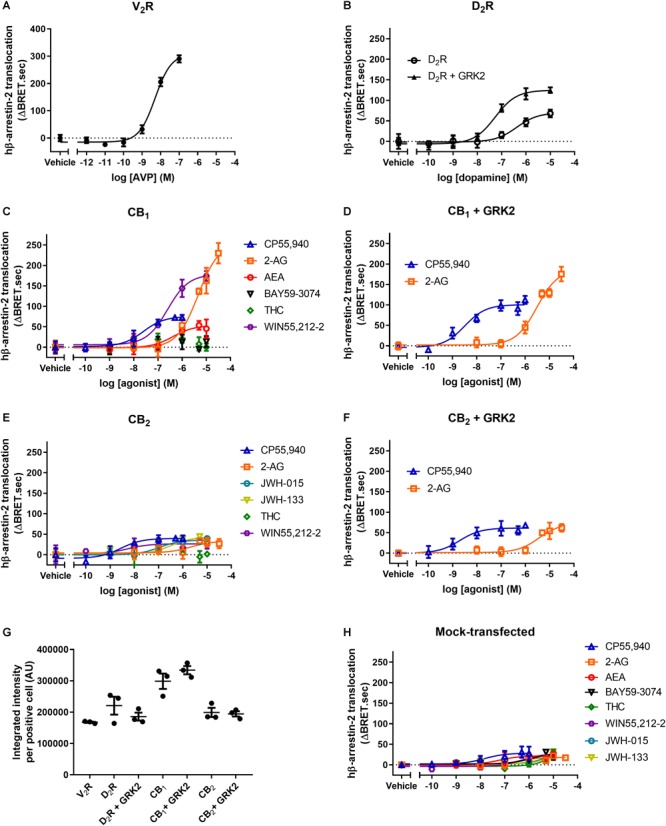
hβ-arrestin-2 translocation to V_2_R, D_2_R, CB_1_, and CB_2_. hβ-Arrestin-2 translocation to plasma membrane in cells expressing **(A)** V_2_R stimulated with AVP, **(B)** D_2_R with or without co-expressed GRK2 stimulated with dopamine, **(C)** CB_1_ stimulated with CP55,940, 2-AG, AEA, BAY, THC, and WIN55,212-2, **(D)** CB_1_ with co-expressed GRK2 stimulated with CP55,940 and 2-AG, **(E)** CB_2_ stimulated with CP55,940, 2-AG, JWH-015, JWH-133, THC, and WIN55,212-2, and **(F)** CB_2_ with co-expressed GRK2 stimulated with CP55,940 and 2-AG. **(G)** Receptor expression of V_2_R, D_2_R, D_2_R+GRK2, CB_1_, CB_1_+GRK2, CB_2_, and CB_2_+GRK2 in cells used in experiments **(A–G)**. Receptor expression was quantified in cells positive for expression by ICC. **(H)** hβ-Arrestin-2 translocation to plasma membrane in mock-transfected cells. Error bars are ±standard deviation of representative data with three technical replicates **(A–F)**, or standard error of the means from three independent biological replicates **(G,H)**.

Bovine β-arrestin-2 has often been used when measuring β-arrestin recruitment to various human GPCRs, presumably due mainly to the convenience of utilizing existing historically available assay constructs. However, given that bovine β-arrestin has been used to make inferences about human physiology, it is necessary to validate that bβ-arrestin-2 functions analogously to the human (h)β-arrestin-2. AVP-induced hβ-arrestin-2 translocation to V_2_R with an equivalent EC50 compared to bβ-arrestin-2 (Figure 2A and Table 3). However, the apparent efficacy of translocation was elevated for hβ-arrestin-2 (ΔBRET.sec ratio 194 versus 306; Figure 2A), possibly reflecting increased translocation of hβ-arrestin-2 in comparison with bβ-arrestin-2, though it is possible that this may be due to differences in biosensor expression or orientation.

**Table 3:** Human β-arrestin-2 translocation to V_2_R and D_2_R.

	hβ-Arrestin-2					
	V_2_R		D_2_R		D_2_R + GRK2	
	logEC50 (±SEM)	Emax (±SEM) (ΔBRET.sec)	logEC50 (±SEM)	Emax (±SEM) (ΔBRET.sec)	logEC50 (±SEM)	Emax (±SEM) (ΔBRET.sec)
AVP	–8.27 ± 0.05	305.9 ± 30.8	X	X	X	X
Dopamine	X	X	–6.40 ± 0.03	72.9 ± 1.5	–7.27 ± 0.04^Δ^	132.0 ± 10.9^Δ^

*X, not determined. ^Δ^Value is significantly different in the presence of GRK2 compared to the absence of GRK2 for the same ligand.*

The translocation of hβ-arrestin-2 to D_2_R was approximately half a log unit less potent than that of bβ-arrestin-2 but the co-expression of GRK2 again significantly increased the efficacy and potency of translocation (both *p* < 0.001, two-way ANOVA) (Figure 2B). Of the six agonists assayed, WIN55,212-2 was the only ligand found to induce translocation of bβ-arrestin-2 and hβ-arrestin-2 with significantly different potencies, being approximately 0.4 log units less potent at translocating bβ-arrestin-2 (*p* = 0.02, two-way ANOVA) (Table 4). The rank order of efficacies was also very similar, with 2-AG the most efficacious, and THC and BAY again failing to translocate the arrestin (Figure 2C) (*p* = 0.059, one-way ANOVA).

**Table 4:** Human β-arrestin-2 translocation to CB_1_ and CB_2_.

	hβ-Arrestin-2							
	CB_1_		CB_1_ + GRK2		CB_2_		CB_2_ + GRK2	
	logEC50 ± SEM (M)	Emax ± SEM (ΔBRET.sec)	logEC50 ± SEM (M)	Emax ± SEM (ΔBRET.sec)	logEC50 ± SEM (M)	Emax ± SEM (ΔBRET.sec)	logEC50 ± SEM (M)	Emax ± SEM (ΔBRET.sec)
CP55,940	–7.81 ± 0.13	83.7 ± 8.6	–8.53 ± 0.03^Δ^	120.0 ± 12.5	–8.70 ± 0.12^§^	37.4 ± 6.2	–8.71 ± 0.06	70.4 ± 7.1^Δ^
2-AG	–5.35 ± 0.09	235.6 ± 17.9	–5.69 ± 0.10^Δ^	149.9 ± 17.6^Δ^	–5.73 ± 0.17	31.3 ± 3.9	–5.68 ± 0.16	68.7 ± 2.5^Δ^
WIN55,212-2	–6.60 ± 0.13^§^	164.2 ± 19.9	X	X	–8.56 ± 0.20^§^	24.4 ± 1.4	X	X
AEA	–6.09 ± 0.19	47.9 ± 3.6	X	X	X	X	X	X
THC	NA^∧^	5.9 ± 3.1^∧^	X	X	NA^∧^	–3.8 ± 4.3^∧^	X	X
BAY	NA^∧^	15.3 ± 4.6^∧^	X	X	X	X	X	X
JWH-015	X	X	X	X	–7.07 ± 0.21	34.0 ± 2.6	X	X
JWH-133	X	X	X	X	–6.55 ± 0.04^§^	37.8 ± 2.3	X	X

*X, Not determined; NA, Not active. ^∧^A concentration–response curve could not be fitted; therefore, efficacy at 10 μM is noted instead of Emax. ^§^Value is significantly different to bovine β-arrestin-2 for the same ligand. ^Δ^Value is significantly different in the presence of GRK2 compared to the absence of GRK2 for the same ligand.*

Co-expression of GRK2 did not alter the efficacy of translocation of hβ-arrestin-2 to CB_1_ when stimulated with CP55,940 (*p* = 0.114, two-way ANOVA), though potency was increased by approximately 0.7 log units (*p* < 0.001, two-way ANOVA) (Figure 2D). Surprisingly, when stimulating with 2-AG, the efficacy was significantly lower when co-expressing GRK2 (149.9 ± 17.6 ΔBRET.sec with GRK2 versus 235.6 ± 17.9 ΔBRET.sec without; *p* = 0.002 two-way ANOVA) (Figure 2D and Table 4), despite similar receptor expression (Figure 2G) and slightly increased potency for inducing translocation (*p* = 0.002, two-way ANOVA).

WIN55,212-2-, JWH-133-, and CP55,940-induced hβ-arrestin-2 translocation to CB_2_ occurred with significantly greater potencies in comparison with bβ-arrestin-2 translocation (*p* < 0.001–0.048, two-way ANOVA) (Figure 2E and Table 4). All ligands tested exhibited similar maximal efficacy to each other, except for THC which was again ineffective (*p* = 0.43, *t*-test).

As was the case for bβ-arrestin-2, when stimulating with either CP55,940 or 2-AG, the efficacy of translocation was significantly enhanced in the presence of co-expressed GRK2 (*p* < 0.001–0.002, two-way ANOVA), however potency was unchanged (*p* = 0.78–0.98, two-way ANOVA) (Figure 2F).

The expression of CB_1_, CB_2_, D_2_R, and V_2_R with or without co-expressed GRK2 was measured by ICC confirming that the co-expression did not affect receptor expression (Figure 2G). Again, all receptors exhibited similar expression levels except CB_1_, which was generally higher than the other receptors.

Off-target hβ-arrestin-2 translocation was assessed in mock-transfected cells. All but one of the cannabinoids produced statistically significant non-CB_1_ or CB_2_-mediated translocation at high concentrations; generally, 10 μM (*p* = 0.001–0.038; except 2-AG, *p* = 0.059), though significant translocation was also evident for CP55,940 at 1 μM and 500 nM (*p* = 0.004, 0.001, respectively; Figure 2H). While the magnitudes of these translocations were small in comparison with some of the robust CB_1_-mediated translocations (namely WIN55,212-2, CP55,940, 2-AG), translocations induced by THC and BAY were equivalent to those measured in CB_1_-expressing cells leading to our conclusion above that these ligands do not induce CB_1_-mediated arrestin translocation. Similarly, for CB_2_, off-target THC response accounted for the entirety of the translocation measured in receptor-transfected cells. For the remainder of the ligands tested at CB_2_, at the highest tested ligand concentration, off-target arrestin translocations were approximately half the maximal measured genuine CB_2_-mediated hβ-arrestin-2 translocation. Neither vasopressin nor dopamine induced measurable off-target hβ-arrestin-2 translocation (data not shown).

### Bovine β-Arrestin-1 (bβ-Arrestin-1) Translocation

**FIGURE 3:**
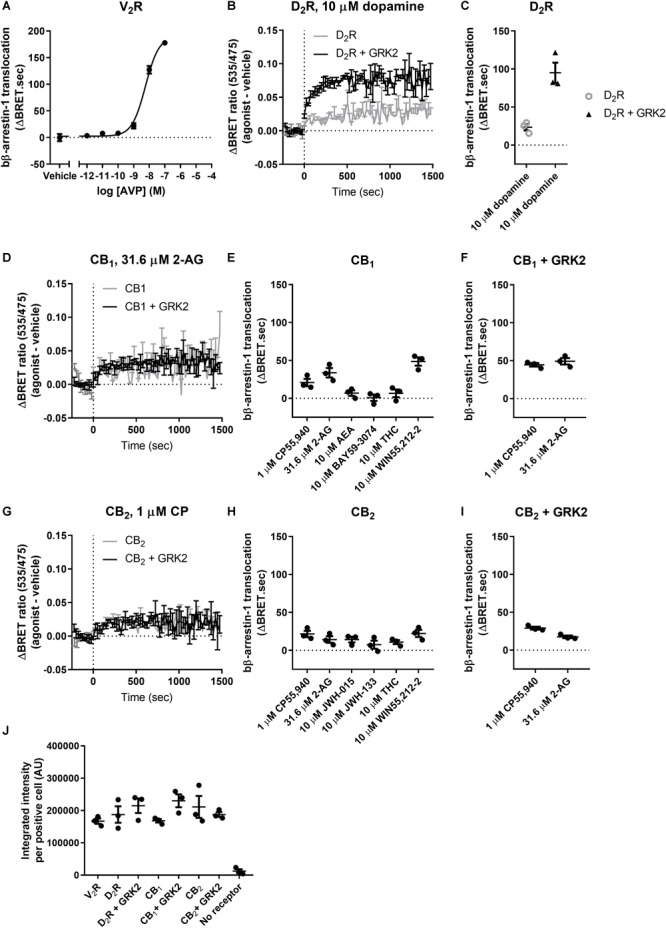
bβ-Arrestin-1 translocation to V_2_R, D_2_R, CB_1_, and CB_2_. **(A)** bβ-Arrestin-1 translocation to V_2_R stimulated with AVP. **(B)** ΔBRET ratio curves of bβ-arrestin-1 translocation to D_2_R with or without co-expressed GRK2 stimulated with 10 μM dopamine. **(C)** AUC quantification of translocation to D_2_R with or without co-expressed GRK2 stimulated with 10 μM dopamine as shown in **(B)**. **(D)** ΔBRET ratio curves of bβ-arrestin-1 translocation to CB_1_ with or without co-expressed GRK2 stimulated with 31.6 μM 2-AG. **(E)** AUC of bβ-arrestin-1 translocation to CB_1_ stimulated with 1 μM CP55,940, 31.6 μM 2-AG, 10 μM AEA, 10 μM BAY, 10 μM THC, and 10 μM WIN55,212-2. **(F)** AUC of bβ-arrestin-1 translocation to CB_1_ with co-expressed GRK2 stimulated with 1 μM CP55,940 and 31.6 μM 2-AG. **(G)** ΔBRET ratio curves of bβ-arrestin-1 translocation to CB_2_ with or without co-expressed GRK2 stimulated with 1 μM CP55,940. **(H)** AUC of bβ-arrestin-1 translocation to CB_2_ stimulated with 1 μM CP55,940, 31.6 μM 2-AG, 10 μM JWH-015, 10 μM JWH-133, 10 μM THC, and 10 μM WIN55,212-2. **(I)** AUC of bβ-arrestin-1 translocation to CB_2_ with co-expressed GRK2 stimulated with 1 μM CP55,940 and 31.6 μM 2-AG. **(J)** Receptor expression of V_2_R, D_2_R, D_2_R+GRK2, CB_1_, CB_1_+GRK2, CB_2_, and CB_2_+GRK2 in cells used in experiments **(A–G)**. Receptor expression was quantified in cells positive for expression by ICC. Error bars represent ±standard deviation of representative data with three technical replicates **(A, B, D, G)** or standard error of the means from three independent biological replicates **(C, E, F, H, I, J)**.

Having investigated the translocation of bovine and human β-arrestin-2, we were interested in comparing these to the translocation of β-arrestin-1. We validated that bβ-arrestin-1 is efficiently translocated to V_2_R with a potency similar to that previously reported (Tenenbaum et al., 2009), confirming that the β-arrestin translocation assay can be used to detect β-arrestin-1 translocation (Table 5 and Figure 3A).

**Table 5:** Bovine β-arrestin-1 translocation to V_2_R and D_2_R.

	bβ-Arrestin-1			
	V_2_R		D_2_R	D_2_R + GRK2
	logEC50 ± SEM (M)	Emax ± SEM (ΔBRET.sec)	Response ± SEM (ΔBRET.sec)	Response ± SEM (ΔBRET.sec)
AVP	–8.237 ± 0.04	176.9 ± 10.9	X	X
10 μM dopamine	X	X	23.4 ± 4.1	95.0 ± 13.3^Δ^

*X, not determined. ^Δ^Value is significantly different in the presence of GRK2 compared to the absence of GRK2 for the same ligand.*

Similarly to β-arrestin-2, the efficacy of translocation of bβ-arrestin-1 to D_2_R was significantly potentiated when co-expressing GRK2 (*p* = 0.001, two-way ANOVA) (Table 5 and Figure 3B,C). Generally, we found that cannabinoids induce little β-arrestin-1 translocation compared to β-arrestin-2 to both CB_1_ and CB_2_ and complete concentration–response curves could not be generated. Hence, we opted to only measure responses with a single high concentration of each agonist. The poor translocation of β-arrestin-1 was not caused by lack of expression of the arrestin, as the Rluc8 signal was comparable to the signal obtained using β-arrestin-2. Utilizing a high concentration of each ligand, only 10 μM WIN55,212-2, 31.6 μM 2-AG, and 1 μM CP55,940 produced statistically significant bβ-arrestin-1 translocation to CB_1_ above vehicle (WIN55,212-2 and 2-AG: *p* < 0.001, CP55,940: *p* = 0.026; one-way ANOVA) (Table 6 and Figure 3D,E). No significant response was observed for the other ligands at CB_1_.

**Table 6:** Bovine β-arrestin-1 translocation to CB_1_ and CB_2_.

	bβ-Arrestin-1			
	CB_1_	CB_1_ + GRK2	CB_2_	CB_2_ + GRK2
	Response ± SEM (ΔBRET.sec)	Response ± SEM (ΔBRET.sec)	Response ± SEM (ΔBRET.sec)	Response ± SEM (ΔBRET.sec)
1 μM CP55,940	20.8 ± 4.9^§^	44.4 ± 2.3^§,Δ^	21.5 ± 4.2^§^	29.0 ± 1.9^§^
31.6 μM 2-AG	33.7 ± 6.2^§^	49.1 ± 4.2^§,Δ^	14.2 ± 4.3	17.5 ± 1.7^§^
10 μM WIN55,212-2	48.5 ± 5.5^§^	X	22.2 ± 4.7^§^	X
10 μM AEA	6.9 ± 3.6	X	X	X
10 μM THC	6.5 ± 5.1	X	10.8 ± 2.6	X
10 μM BAY	0.7 ± 4.2	X	X	X
10 μM JWH-015	X	X	14.2 ± 3.7	X
10 μM JWH-133	X	X	7.4 ± 5.3	X

*X, not determined. ^§^Value is significantly different from vehicle control. ^Δ^Value is significantly different in the presence of GRK2 compared to the absence of GRK2 for the same ligand.*

The co-expression of GRK2 significantly elevated the translocation of bβ-arrestin-1 after stimulation with both CP55,940 and 2-AG (CP55,940: *p* = 0.007, 2-AG: *p* = 0.046, two-way ANOVA), although the change in efficacy was considerably smaller than that conferred via D_2_R (Table 6 and Figure 3D,F).

For CB_2_, the translocation of bβ-arrestin-1 is weak but WIN55,212-2 and CP55,940 stimulated translocation significantly differently from vehicle (Both: *p* = 0.008, one-way ANOVA; Figure 3G,H). Co-expression of GRK2 did not alter the response significantly (*p* = 0.137), though a slightly greater mean response and smaller between-experiment variability resulted in 2-AG inducing a measurable bβ-arrestin-1 translocation in the presence of GRK2, which was not detected when GRK2 was absent (with GRK2: *p* < 0.001, without GRK2: *p* = 0.088; Table 6 and Figure 3G,I).

The receptor expression was equivalent between all the experiments involving bβ-arrestin-1 as measured by ICC (Figure 3J). As per our procedure for hβ-arrestin-2, off-target bβ-arrestin-1 translocation was assessed in mock-transfected cells. Only 10 μM JWH-015 induced a detectable signal above the vehicle control (22.4 ± 4.3 ΔBRET.sec; *p* = 0.005, one-way ANOVA).

### Human β-Arrestin-1 (hβ-Arrestin-1) Translocation

**FIGURE 4:**
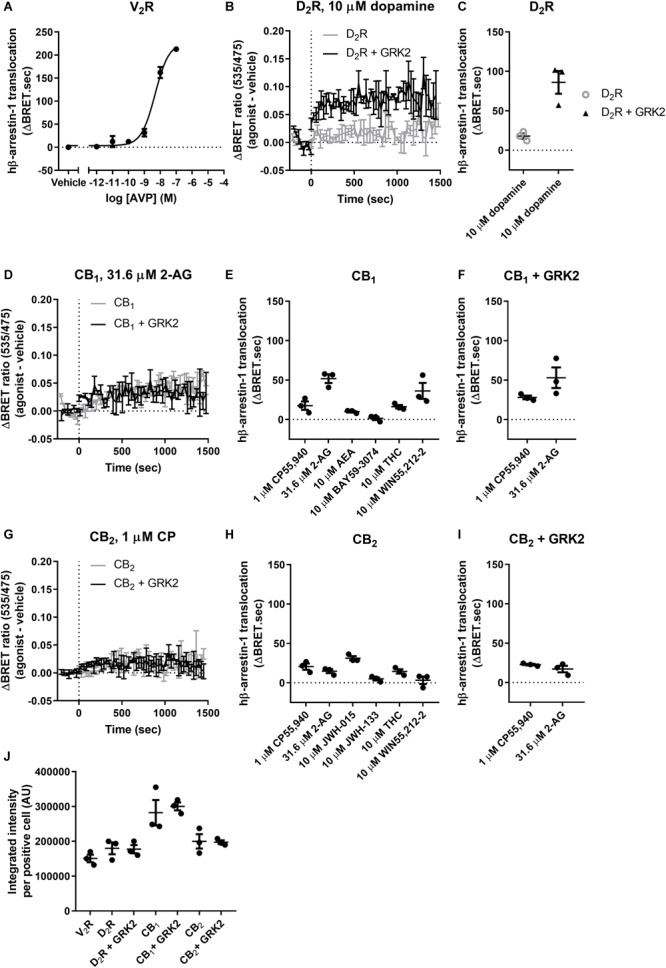
hβ-Arrestin-1 translocation to V_2_R, D_2_R, CB_1_, and CB_2_. **(A)** hβ-Arrestin-1 translocation to V_2_R stimulated with AVP. **(B)** ΔBRET ratio curves of hβ-arrestin-1 translocation to D_2_R with or without co-expressed GRK2 stimulated with 10 μM dopamine. **(C)** AUC quantification of translocation to D_2_R with or without co-expressed GRK2 stimulated with 10 μM dopamine as shown in **B**. **(D)** ΔBRET ratio curves of hβ-arrestin-1 translocation to CB_1_ with or without co-expressed GRK2 stimulated with 31.6 μM 2-AG. **(E)** AUC of hβ-arrestin-1 translocation to CB_1_ stimulated with 1 μM CP55,940, 31.6 μM 2-AG, 10 μM AEA, 10 μM BAY, 10 μM THC, and 10 μM WIN55,212-2. **(F)** AUC of hβ-arrestin-1 translocation to CB_1_ with co-expressed GRK2 stimulated with 1 μM CP55,940 and 31.6 μM 2-AG. **(G)** ΔBRET ratio curves of hβ-arrestin-1 translocation to CB_2_ with or without co-expressed GRK2 stimulated with 1 μM CP. **(H)** AUC of hβ-arrestin-1 translocation to CB_2_ stimulated with 1 μM CP55,940, 31.6 μM 2-AG, 10 μM JWH-015, 10 μM JWH-133, 10 μM THC, and 10 μM WIN55,212-2. **(I)** AUC of hβ-arrestin-1 translocation to CB_2_ with co-expressed GRK2 stimulated with 1 μM CP55,940 and 31.6 μM 2-AG. **(J)** Receptor expression of V_2_R, D_2_R, D_2_R+GRK2, CB_1_, CB_1_+GRK2, CB_2_, and CB_2_+GRK2 in cells used in experiments **(A–G)**. Receptor expression was quantified in cells positive for expression by ICC. Error bars represent ±standard deviation of representative data with three technical replicates **(A, B, D, G)** or standard error of the means from three independent biological replicates **(C, E, F, H, I, J)**.

We wanted to compare our findings with bβ-arrestin-1 to hβ-arrestin-1. Again, we used V_2_R to validate the translocation of hβ-arrestin-1 and this was indeed recruited with similar efficacy and potency as bβ-arrestin-1 (Figure 4A). As was also expected from our bβ-arrestin-1 data, the translocation of hβ-arrestin-1 to D_2_R was significantly potentiated when GRK2 was co-expressed (*p* = 0.001, two-way ANOVA) (Table 7 and Figure 4B,C).

**Table 7:** Human β-arrestin-1 translocation to V_2_R and D_2_R.

	hβ-Arrestin-1			
	V_2_R		D_2_R	D_2_R + GRK2
	logEC50 ± SEM (M)	Emax ± SEM (ΔBRET.sec)	Response ± SEM (ΔBRET.sec)	Response ± SEM (ΔBRET.sec)
AVP	–8.33 ± 0.03	246.3 ± 21.8	X	X
10 μM dopamine	X	X	17.9 ± 3.3	86.1 ± 14.5^Δ^

*X, not determined. ^Δ^Value is significantly different in the presence of GRK2 compared to the absence of GRK2 for the same ligand.*

Also in contrast to bβ-arrestin-1, the co-expression of GRK2 did not significantly alter the translocation of hβ-arrestin-1 after stimulation with CP55,940 or 2-AG (*p* = 0.467), although a slightly greater mean response and smaller between-experiment variability resulted in CP55,940 inducing a measurable bβ-arrestin-1 translocation in the presence of GRK2 (*p* = 0.008; Table 8 and Figure 4D,F).

**Table 8:** Human β-arrestin-1 translocation to CB_1_ and CB_2_.

	hβ-Arrestin-1?			
	CB_1_	CB_1_ + GRK2	CB_2_	CB_2_ + GRK2
	Response ± SEM (ΔBRET.sec)	Response ± SEM (ΔBRET.sec)	Response ± SEM (ΔBRET.sec)	Response ± SEM (ΔBRET.sec)
1 μM CP55,940	17.6 ± 5.3	28.1 ± 2.3^§^	20.5 ± 3.9^§^	22.7 ± 1.1^§^
31.6 μM 2-AG	51.8 ± 5.6^§^	53.0 ± 13.1^§^	14.7 ± 2.5^§^	17.2 ± 4.2^§^
10 μM WIN55,212-2	36.2 ± 10.2^§^	X	3.2 ± 4.7	X
10 μM AEA	10.0 ± 1.0	X	X	X
10 μM THC	16.2 ± 2.2	X	14.4 ± 2.8^§^	X
10 μM BAY	1.4 ± 2.1	X	X	X
10 μM JWH-015	X	X	31.2 ± 2.6^§^	X
10 μM JWH-133	X	X	4.9 ± 2.0	X

*X, not determined. ^§^Value is significantly different from vehicle control. ^Δ^Value is significantly different in the presence of GRK2 compared to the absence of GRK2 for the same ligand.*

For CB_2_, hβ-arrestin-1 translocation was significantly different from vehicle when stimulating with 10 μM JWH-015, 1 μM CP55,940, 31.6 μM 2-AG, and 10 μM THC (*p* = 0.001–0.012; Table 8 and Figure 4H) but not for 10 μM WIN55,212-2 or JWH-133 (both: *p* = 0.46), however, generally the translocation is weak and not affected by the co-expression of GRK2 (*p* = 0.48; Figure 4G,I). Receptor expression was confirmed by ICC (Figure 4J), and no ligand-induced hβ-arrestin-1 translocation was detected in mock-transfected cells (*p* = 0.13, one-way ANOVA).

## Discussion

This study set out to systematically characterize the ability of a structurally diverse range of ligands to drive the translocation of β-arrestin-1 and -2 through CB stimulation. We have further extended the study to investigate whether bovine and human arrestins function equivalently in this assay. To this end we utilized a BRET assay monitoring arrestin translocation to the plasma membrane that does not require modification of the receptor(s) of interest, initially developed to investigate interactions between D_2_R and arrestins (Clayton et al., 2014; Donthamsetti et al., 2015). We have thus utilized D_2_R and the robust arrestin recruiter V_2_R (Oakley et al., 1999; Charest and Bouvier, 2003) as controls to validate the assay in our laboratory.

Initial studies focused on the ability of CB_1_ activation to drive the translocation of bovine β-arrestin-2 in response to a structurally diverse range of agonists. The results showed a wide range of agonist efficacies, with the endogenous agonist 2-AG being the most efficacious, whereas the phytocannabinoid THC and partial synthetic agonist BAY did not significantly activate this pathway. The rank order of potencies for the effective ligands was CP55,940 >> WIN55,212-2 > AEA, 2-AG.

We further sought to investigate whether the recruitment mediated by CBRs was similar between bovine and human arrestins. The relative ability of ligands to induce arrestin translocation in the presence of hCB_1_ expression was generally very similar between bovine and human β-arrestin-2, with the same rank order of efficacy. WIN55,212 was moderately (approximately half a log unit) more potent in recruiting bovine than human β-arrestin 2.

Similarly, to bβ-arrestin-1, the translocation of hβ-arrestin-1 was significantly different from vehicle only when stimulating CB_1_ with 2-AG and WIN55,212-2 (both: *p* < 0.001, one-way ANOVA), however, unlike the bovine counterpart CP55,940 did not induce translocation of hβ-arrestin-1 to a statistically measurable degree (*p* = 0.097; Table 8 and Figure 4D,E).

We recently characterized the same group of ligands as studied here for CB_1_-mediated arrestin translocation in the context of modulation of cAMP production via CB_1_ (Finlay et al., 2017). As well as inhibiting the production of cAMP via Gα_i_, CB_1_ is known to also couple to the stimulation of cAMP through a putative Gα_s_ mechanism under some circumstances (Glass and Felder, 1997; Finlay et al., 2017). It is interesting to note that the ligands’ potencies for inducing β-arrestin-2 recruitment at CB_1_ are well correlated with their potencies for driving increases in cAMP (as revealed when cells were pertussis toxin-treated), whereas Gα_i_-mediated cAMP inhibition was induced with between 10 and 100-fold greater potency. The low potency for inducing β-arrestin-2 recruitment is unlikely to be due to low receptor expression as the transient transfection of pplss-tagged hCB_1_ is known to result in high expression levels (Finlay et al., 2017). Furthermore, we previously observed a similar pattern and rank order of efficacy for Gα_s_-mediated stimulation of cAMP synthesis as seen here for β-arrestin-2 recruitment (including lack of efficacy for THC and BAY, and moderate efficacy for CP55,940 which is widely assumed to be a full agonist), whereas all ligands were similarly effective at inhibiting cAMP production via Gα_i_.

These observations are consistent with CB_1_ having a low relative efficiency for coupling to both the Gα_s_ and β-arrestin-2 effector pathways in comparison with Gα_i_. While we have not interrogated this directly, perhaps the CB_1_ receptor conformational state(s) requisite for β-arrestin-2 recruitment are more closely related to those required for Gα_s_ activation, than for Gα_i_. We are also aware that our model system (the CB_1_-expressing HEK cell line and associated assays utilized) exhibits “receptor reserve” for the Gα_i_ pathway, wherein the system’s maximal response is reached at low receptor occupancy, and the potential ability to exert greater efficacy via occupation of more receptors is manifested as increases in potency because the system maximum effect has already been reached. In comparison, perhaps the Gα_s_ and β-arrestin-2 pathways are able to report a relatively greater dynamic range of efficacy, and thereby the efficacies and potencies indicate more directly the true intrinsic efficacy (“stimulus per receptor”) of the ligands (Kenakin, 2013). Indeed, it has been suggested that β-arrestin recruitment is less likely than downstream pathways to be subject to receptor reserve due to less signal amplification (Smith and Rajagopal, 2016).

The maximum observed efficacy of CB_1_ β-arrestin-2 recruitment was substantially lower than that generated by V_2_R, but was similar to the efficacy of D_2_R in recruiting β-arrestin-2 in response to dopamine. In the case of D_2_R, co-expression of GRK2 has been shown to enhance the potency and efficacy of this interaction (Clayton et al., 2014), a finding we were able to replicate, and thus we investigated if GRK2 expression could also enhance the ability of the cannabinoid ligands to drive this pathway. In contrast to D_2_R however, the co-expression of GRK2 did not enhance the efficacy of the response to either of the cannabinoids tested (CP55,940 and 2-AG) but did enhance the potency of the ligands (although this only reached statistical significance for CP55,940). This relatively subtle change was surprising, as previous studies have suggested an interaction between GRK2, β-arrestin-2, and CB_1_. For example, the presynaptic expression of dominant negative GRK2 or β-arrestin-2 reduced desensitization of CB_1_ receptor-mediated presynaptic inhibition of glutamatergic neurotransmission in rat hippocampal neurons (Kouznetsova et al., 2002). This could reflect that the endogenous level of GRK2 expression in these cells is sufficient for CB_1_-mediated interactions with bβ-arrestin-2, although the pronounced effect of GRK2 on dopaminergic signaling would suggest that this is not the case. The endogenous expression of GRKs in HEK cells is not clear; Atwood et al. (2011) reported GRK3-5 were expressed but not GRK1 or 2, however two previous papers had identified GRK2 in these cells (Hasbi et al., 2004; Zidar et al., 2009).

The inability to detect an hCB_1_-mediated response to THC in this assay was also surprising in light of prior reports. THC has been shown to produce greater antinociception and hypothermia in β-arrestin-2^(-/-)^ mice compared to wild-type mice, yet no differences were observed in either assay for CP55,940 (Breivogel et al., 2008). Meanwhile, two *in vitro* studies have suggested that THC induces β-arrestin-2 recruitment via CB_1_, though with considerably different reported potencies (Soethoudt et al., 2017; Navarro et al., 2018). The disparity in findings may reflect differential GRK or other adaptor protein [such as CRIP1a (Blume et al., 2017)] expression between cell types and lines; Soethoudt et al. (2017) performed their assays on Chinese Hamster Ovary (CHO) cells and Navarro et al. (2018) utilized the HEK-293 “T” subclone, which is known to harbor considerable genomic differences in comparison with the parental HEK-293 line which we utilized in our experiments (Lin et al., 2014). Further studies could address the dependence of THC-stimulated arrestin recruitment on various GRK isoforms and arrestin- and/or CB_1_-associated signaling adaptor proteins. If this were the case, it would imply that THC induces a substantially different conformation of the receptor, which in turn is phosphorylated differently to the other agonists.

It is also important to consider differences which may have arisen due to the arrestin assays used; both Soethoudt et al. (2017) and Navarro et al. (2018) monitored translocation of tagged arrestin to tagged receptor. The former study utilized a PathHunter assay, which calls for a “ProLink tagged receptor,” and thus we wonder whether changes in assay signal may be at least partially reflective of a change in conformation or activation of the receptor rather than the actual capacity of the native receptor to recruit arrestin. Another comparative advantage of our approach, in which arrestin translocation to the plasma membrane is measured without the requirement for the receptor of interest to be tagged, is the ability to carry out assays in the absence of receptor to determine whether a portion of the signal is not mediated by the receptor of interest. The source of this non-specific signal is not clear, but with all fluorescent/luminescent proteins it is possible that changes in conformation could result in quenching or unquenching of the signal produced. With cannabinoid ligands being highly lipophilic and able to cross the plasma membrane there is more potential for this to occur than with other non-cell-permeable small molecules. Importantly, non-CB_1_/CB_2_-mediated effects of cannabinoids may occur via other putative CBRs and other targets, particularly when applied at high concentrations (Felder et al., 1992; Soethoudt et al., 2017). All the receptor-mediated arrestin translocation data shown in this paper is following the subtraction of the non-receptor-of-interest mediated component of the signal; the measured non-receptor-mediated signals are shown separately. Although these non-CB-mediated responses were low potency relative to the receptor-mediated responses, these have clear potential to influence the apparent receptor-mediated concentration–response curve if not accounted for. Tellingly, the entire β-arrestin-2 response we observed for THC or BAY appeared to be non-CB_1/2_-mediated. Thus, the difference between our findings and those described above could also be due to the inability of prior assays to distinguish between receptor and non-receptor-mediated signal.

While the presence versus absence of THC response was the most striking difference in finding in comparison with prior *in vitro* studies, for effective agonists we also observed some differences in rank maximum efficacy; our data indicated 2-AG > WIN55,212-2 > CP55,940, whereas Soethoudt et al. (2017) observed CP55,940, WIN55,212-2 > 2-AG, despite measured potencies being similar. We can only speculate that a combination of mechanisms described above may contribute to the differences observed. The possibility that differential expression of signaling adaptors might give rise to completely altered CB_1_-mediated arrestin recruitment patterns is intriguing and is indicative of system factors influencing the measurement of ligand bias. This also reinforces that when studying ligand bias it is important to perform the various signaling assays for comparison under as closely analogous conditions as possible.

We continued by examining a range of ligands at hCB_2_ for bovine and human β-arrestin-2. All ligands tested except THC were able to translocate bovine and human β-arrestin-2, although in general the maximum efficacy for all ligands was low. Interestingly, bovine β-arrestin-2 was translocated with greater efficacy (relative to V_2_R and CB_1_) than human β-arrestin-2; however, human β-arrestin-2 responses were more potent for three of the five agonists with measurable responses.

Our finding of a lack of efficacy for THC again differs from Soethoudt et al. (2017), wherein THC was able to recruit β-arrestin-2 to CB_2_ utilizing a PathHunter assay in CHO cells. However, the response reported was only partial and did not reach a plateau by 10 μM. Furthermore, an earlier study reported measurable but extremely slight THC-induced arrestin recruitment; again, a concentration–response curve could not be drawn (Dhopeshwarkar and Mackie, 2016). We suspect a considerable contributor to the differences between these studies is whether or not non-receptor-of-interest-mediated arrestin assay signal was controlled for. However, other than THC, our relative CB_2_ efficacy results align very closely with Soethoudt et al. (2017) and an earlier study which included a smaller subset of ligands in HEK cells with the PathHunter assay system (McGuinness et al., 2009). In contrast, a 2016 study (Dhopeshwarkar and Mackie, 2016) of mouse CB_2_ in HEK cells could not detect 2-AG induced β-arrestin-2 recruitment and measured relatively lower efficacy responses for WIN55,212-2, JWH-015, and JWH-133. We suggest the disparity of the latter study with ours and the other two noted studies were likely due to the utilization of rodent CB_2_; indeed, important ligand binding and signaling disparities between mouse, rat, and human CB_2_ have been reported (Griffin et al., 2000; Mukherjee et al., 2004).

In contrast to β-arrestin-2, very few cannabinoid ligands generated a statistically significant bovine β-arrestin-1 response through either CB_1_ (WIN55,212-2 > 2-AG > CP55,940) or CB_2_ (WIN55,212-2, CP55,940), despite robust translocation induced by V_2_R. Controls for receptor expression suggested very similar expression of CB_1_ and CB_2_ relative to these receptors, thus it seems unlikely that the low efficacy of responses was due to insufficient receptor expression. The small response window prevented reliable assessment of concentration–response curves and therefore the data reported are for the response generated at maximal tested concentrations. Similarly, only 2-AG and WIN55,212-2 induced hβ-arrestin-1 translocation to CB_1_ significantly differently from vehicle. In contrast to bβ-arrestin-1, the translocation of hβ-arrestin-1 to CB_2_ could be significantly induced by JWH-015, CP55,940, 2-AG, and THC. Interestingly, this was the only condition in our study under which we detected CB-mediated THC-induced arrestin translocation. Our observations are consistent with a weak interaction between β-arrestin-1 and CB_1_ and CB_2_ as reported by some (Gyombolai et al., 2013, 2015) and corroborated by the fact that β-arrestin-2, but not β-arrestin-1, could not be immunoprecipitated with CB_1_ (Delgado-Peraza et al., 2016). Nonetheless, a direct interaction between β-arrestin-1 and CB_1_, and β-arrestin-1-mediated signaling have been observed in some studies, although most notably with non-orthosteric ligands (Laprairie et al., 2014; Delgado-Peraza et al., 2016; Laprairie et al., 2016). It is therefore possible that β-arrestin-1 has only weak affinity for CB_1_ and CB_2_ or is only very transiently interacting with these receptors, and therefore not efficiently captured by this assay. Complementation assays may be more sensitive to very transient signals as they are non-reversible, essentially acting as an accumulation assay. We also note the possibility that β-arrestin-1 could bind with a different modality/conformation to CB_1_ and CB_2_ versus V_2_R and D_2_R which may be sufficient to reduce BRET signal even though translocation may still be present. Although we feel that this likelihood is small, it would be interesting to carry out imaging experiments to test this possibility. GRK2 potentiated the translocation of both species of β-arrestin-1 to D_2_R, but CB_1_ and CB_2_ β-arrestin-1 translocation was only enhanced very slightly.

Although previously used in connection with D_2_R, we have optimized a receptor-independent bβ-arrestin-2 translocation BRET assay for CB_1_, CB_2_, and V_2_R and added tools for measuring bβ-arrestin-1 and human β-arrestin-1 and 2. This assay represents a new tool for drug discovery which is amenable to high-throughput screening and is therefore particularly useful for discovery and characterization of biased ligands targeting GPCRs. Given the conflicts already in the literature and findings presented here, this work highlights that there can be different outcomes between widely used β-arrestin recruitment assays and the non-receptor-tagged translocation assay used for this work. These disparities are likely due to system factors, such as co-expression of requisite accessory proteins and whether or not non-receptor-of-interest arrestin assay signal is appropriately controlled. Furthermore, this study has revealed potentially important differences between utilization of bovine versus human arrestins. These issues should be taken into consideration by future studies investigating arrestin recruitment to CBRs to avoid misplaced conclusions.

## Author Contributions

MI designed and performed the majority of the experiments, and wrote the first draft of the manuscript. DF designed and made the human arrestin constructs and contributed to the data analysis. MP contributed experimental datasets. JJ provided the arrestin assay and technical expertise. MI, MG, and NG conceived of the experiments. MG and NG oversaw all elements of the project, obtained the funding, and contributed to experimental design and analysis. All authors reviewed and edited the manuscript.

## Conflict of Interest Statement

Columbia University has applied for a patent for a related arrestin translocation assay, and the technology has been licensed; JJ is an inventor. In all other respects, the authors declare that the research was conducted in the absence of any commercial or financial relationships that could be construed as a potential conflict of interest. The remaining authors declare that the research was conducted in the absence of any commercial or financial relationships that could be construed as a potential conflict of interest.
